# Globalization and Economic Growth: Empirical Evidence on the Role of Complementarities

**DOI:** 10.1371/journal.pone.0087824

**Published:** 2014-04-10

**Authors:** Parisa Samimi, Hashem Salarzadeh Jenatabadi

**Affiliations:** 1 Faculty of Management, Universiti Teknologi Malaysia (UTM), Johor, Malaysia; 2 Department of Management, Mobarakeh Branch, Islamic Azad University, Isfahan, Iran; 3 Applied Statistics Department, Economics and Administration Faculty, University of Malaya, Kuala Lumpur, Malaysia; Cinvestav-Merida, Mexico

## Abstract

This study was carried out to investigate the effect of economic globalization on economic growth in OIC countries. Furthermore, the study examined the effect of complementary policies on the growth effect of globalization. It also investigated whether the growth effect of globalization depends on the income level of countries. Utilizing the generalized method of moments (GMM) estimator within the framework of a dynamic panel data approach, we provide evidence which suggests that economic globalization has statistically significant impact on economic growth in OIC countries. The results indicate that this positive effect is increased in the countries with better-educated workers and well-developed financial systems. Our finding shows that the effect of economic globalization also depends on the country’s level of income. High and middle-income countries benefit from globalization whereas low-income countries do not gain from it. In fact, the countries should receive the appropriate income level to be benefited from globalization. Economic globalization not only directly promotes growth but also indirectly does so via complementary reforms.

## Introduction

Globalization, as a complicated process, is not a new phenomenon and our world has experienced its effects on different aspects of lives such as economical, social, environmental and political from many years ago [1]–[4]. Economic globalization includes flows of goods and services across borders, international capital flows, reduction in tariffs and trade barriers, immigration, and the spread of technology, and knowledge beyond borders. It is source of much debate and conflict like any source of great power.

**Figure 1:**
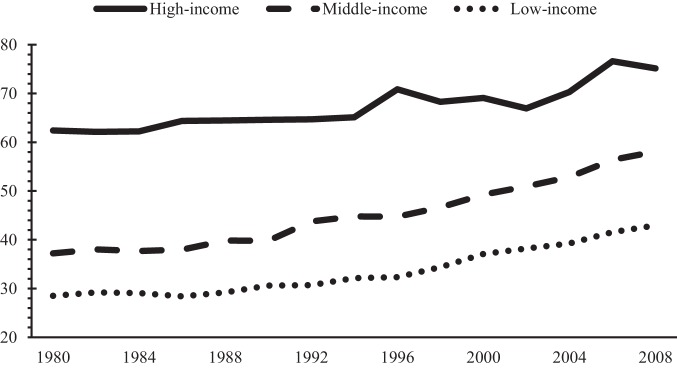
Average economic globalization (KOF index) by income groups.

The broad effects of globalization on different aspects of life grab a great deal of attention over the past three decades. As countries, especially developing countries are speeding up their openness in recent years the concern about globalization and its different effects on economic growth, poverty, inequality, environment and cultural dominance are increased. As a significant subset of the developing world, Organization of Islamic Cooperation (OIC) countries are also faced by opportunities and costs of globalization. Figure 1 shows the upward trend of economic globalization among different income group of OIC countries.

Although OICs are rich in natural resources, these resources were not being used efficiently. It seems that finding new ways to use the OICs economic capacity more efficiently are important and necessary for them to improve their economic situation in the world. Among the areas where globalization is thought, the link between economic growth and globalization has been become focus of attention by many researchers. Improving economic growth is the aim of policy makers as it shows the success of nations. Due to the increasing trend of globalization, finding the effect of globalization on economic growth is prominent.

The net effect of globalization on economic growth remains puzzling since previous empirical analysis did not support the existent of a systematic positive or negative impact of globalization on growth. Most of these studies suffer from econometrics shortcoming, narrow definition of globalization and small number of countries. The effect of economic globalization on the economic growth in OICs is also ambiguous. Existing empirical studies have not indicated the positive or negative impact of globalization in OICs. The relationship between economic globalization and economic growth is important especially for economic policies.

Recently, researchers have claimed that the growth effects of globalization depend on the economic structure of the countries during the process of globalization. The impact of globalization on economic growth of countries also could be changed by the set of complementary policies such as improvement in human capital and financial system. In fact, globalization by itself does not increase or decrease economic growth. The effect of complementary policies is very important as it helps countries to be successful in globalization process.

In this paper, we examine the relationship between economic globalization and growth in panel of selected OIC countries over the period 1980–2008. Furthermore, we would explore whether the growth effects of economic globalization depend on the set of complementary policies and income level of OIC countries.

The paper is organized as follows. The next section consists of a review of relevant studies on the impact of globalization on growth. Afterward the model specification is described. It is followed by the methodology of this study as well as the data sets that are utilized in the estimation of the model and the empirical strategy. Then, the econometric results are reported and discussed. The last section summarizes and concludes the paper with important issues on policy implications.

## Literature Review

The relationship between globalization and growth is a heated and highly debated topic on the growth and development literature. Yet, this issue is far from being resolved. Theoretical growth studies report at best a contradictory and inconclusive discussion on the relationship between globalization and growth. Some of the studies found positive the effect of globalization on growth through effective allocation of domestic resources, diffusion of technology, improvement in factor productivity and augmentation of capital [5], [6]. In contrast, others argued that globalization has harmful effect on growth in countries with weak institutions and political instability and in countries, which specialized in ineffective activities in the process of globalization [5], [7], [8].

Given the conflicting theoretical views, many studies have been empirically examined the impact of the globalization on economic growth in developed and developing countries. Generally, the literature on the globalization-economic growth nexus provides at least three schools of thought. First, many studies support the idea that globalization accentuates economic growth [9]–[19]. Pioneering early studies include Dollar [9], Sachs et al. [15] and Edwards [11], who examined the impact of trade openness by using different index on economic growth. The findings of these studies implied that openness is associated with more rapid growth.

In 2006, Dreher introduced a new comprehensive index of globalization, KOF, to examine the impact of globalization on growth in an unbalanced dynamic panel of 123 countries between 1970 and 2000. The overall result showed that globalization promotes economic growth. The economic and social dimensions have positive impact on growth whereas political dimension has no effect on growth. The robustness of the results of Dreher [19] is approved by Rao and Vadlamannati [20] which use KOF and examine its impact on growth rate of 21 African countries during 1970–2005. The positive effect of globalization on economic growth is also confirmed by the extreme bounds analysis. The result indicated that the positive effect of globalization on growth is larger than the effect of investment on growth.

The second school of thought, which supported by some scholars such as Alesina et al. [21], Rodrik [22] and Rodriguez and Rodrik [23], has been more reserve in supporting the globalization-led growth nexus. Rodriguez and Rodrik [23] challenged the robustness of Dollar (1992), Sachs, Warner et al. (1995) and Edwards [11] studies. They believed that weak evidence support the idea of positive relationship between openness and growth. They mentioned the lack of control for some prominent growth indicators as well as using incomprehensive trade openness index as shortcomings of these works. Warner [24] refuted the results of Rodriguez and Rodrik (2000). He mentioned that Rodriguez and Rodrik (2000) used an uncommon index to measure trade restriction (tariffs revenues divided by imports). Warner (2003) explained that they ignored all other barriers on trade and suggested using only the tariffs and quotas of textbook trade policy to measure trade restriction in countries.

Krugman [25] strongly disagreed with the argument that international financial integration is a major engine of economic development. This is because capital is not an important factor to increase economic development and the large flows of capital from rich to poor countries have never occurred. Therefore, developing countries are unlikely to increase economic growth through financial openness. Levine [26] was more optimistic about the impact of financial liberalization than Krugman. He concluded, based on theory and empirical evidences, that the domestic financial system has a prominent effect on economic growth through boosting total factor productivity. The factors that improve the functioning of domestic financial markets and banks like financial integration can stimulate improvements in resource allocation and boost economic growth.

The third school of thoughts covers the studies that found nonlinear relationship between globalization and growth with emphasis on the effect of complementary policies. Borensztein, De Gregorio et al. (1998) investigated the impact of FDI on economic growth in a cross-country framework by developing a model of endogenous growth to examine the role of FDI in the economic growth in developing countries. They found that FDI, which is measured by the fraction of products produced by foreign firms in the total number of products, reduces the costs of introducing new varieties of capital goods, thus increasing the rate at which new capital goods are introduced. The results showed a strong complementary effect between stock of human capital and FDI to enhance economic growth. They interpreted this finding with the observation that the advanced technology, brought by FDI, increases the growth rate of host economy when the country has sufficient level of human capital. In this situation, the FDI is more productive than domestic investment.

Calderón and Poggio [27] examined the structural factors that may have impact on growth effect of trade openness. The growth benefits of rising trade openness are conditional on the level of progress in structural areas including education, innovation, infrastructure, institutions, the regulatory framework, and financial development. Indeed, they found that the lack of progress in these areas could restrict the potential benefits of trade openness. Chang et al. [28] found that the growth effects of openness may be significantly improved when the investment in human capital is stronger, financial markets are deeper, price inflation is lower, and public infrastructure is more readily available. Gu and Dong [29] emphasized that the harmful or useful growth effect of financial globalization heavily depends on the level of financial development of economies. In fact, if financial openness happens without any improvement in the financial system of countries, growth will replace by volatility.

However, the review of the empirical literature indicates that the impact of the economic globalization on economic growth is influenced by sample, econometric techniques, period specifications, observed and unobserved country-specific effects. Most of the literature in the field of globalization, concentrates on the effect of trade or foreign capital volume (de facto indices) on economic growth. The problem is that de facto indices do not proportionally capture trade and financial globalization policies. The rate of protections and tariff need to be accounted since they are policy based variables, capturing the severity of trade restrictions in a country. Therefore, globalization index should contain trade and capital restrictions as well as trade and capital volume. Thus, this paper avoids this problem by using a comprehensive index which called KOF [30]. The economic dimension of this index captures the volume and restriction of trade and capital flow of countries.

Despite the numerous studies, the effect of economic globalization on economic growth in OIC is still scarce. The results of recent studies on the effect of globalization in OICs are not significant, as they have not examined the impact of globalization by empirical model such as Zeinelabdin [31] and Dabour [32]. Those that used empirical model, investigated the effect of globalization for one country such as Ates [33] and Oyvat [34], or did it for some OIC members in different groups such as East Asia by Guillaumin [35] or as group of developing countries by Haddad et al. [36] and Warner [24]. Therefore, the aim of this study is filling the gap in research devoted solely to investigate the effects of economic globalization on growth in selected OICs. In addition, the study will consider the impact of complimentary polices on the growth effects of globalization in selected OIC countries.

## Model Specification

This study uses a dynamic panel data model to investigate the effect of globalization on economic growth. The model can be shown as follows:
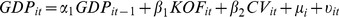
(1)where i is country index, t is time index, 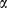 and 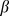 are the parameters to be estimated, GDP is the logarithm of real GDP per capita, KOF is economic globalization, CV is a vector of other control variables that affect economic growth, 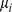 is unobserved country-specific effect term, and 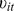 is the usual error term. The group of control variables is comprised of variables frequently used in the growth literature including government consumption, secondary school enrolment as a proxy for human capital, inflation (consumer price index), domestic investment, liquid liability to capture the financial development and ICRG as an index for institutional quality.

## Methodology and Data

In Eq.1, the existence of lag per capita GDP produces the well-known dynamic panel bias due to the correlation between the 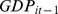 and disturbance term, 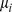. In other words, 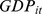 is a function of 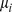, as 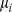 is time-invariant, therefore, 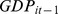 is also a function of 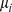. It means that Eq. 1 has a severe endogeneity problem that happens when the lag of dependent variable, as one of the regressors, is correlated with one component of the error term [37]. In addition, In Eq.1, the fixed effects or time-invariant country characteristics (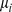), might be correlated with the explanatory variables which violates the assumptions underlying the classical linear regression model. In this case, the simple ordinary least squares (OLS) or fixed and random effects approaches can produce highly misleading results.

This paper applies the generalized method of moments (GMM) panel estimator first suggested by Anderson and Hsiao [38] and later developed further by Arellano and Bond [39]. This flexible method requires only weak assumption that makes it one of the most widely used econometric techniques especially in growth studies. The dynamic GMM procedure is as follow: first, to eliminate the individual effect form dynamic growth model, the method takes differences. Then, it instruments the right hand side variables by using their lagged values. The last step is to eliminate the inconsistency arising from the endogeneity of the explanatory variables.

The consistency of the GMM estimator depends on two specification tests. The first is a Sargan test of over-identifying restrictions, which tests the overall validity of the instruments. Failure to reject the null hypothesis gives support to the model. The second test examines the null hypothesis that the error term is not serially correlated.

The GMM can be applied in one- or two-step variants. The one-step estimators use weighting matrices that are independent of estimated parameters, whereas the two-step GMM estimator uses the so-called optimal weighting matrices in which the moment conditions are weighted by a consistent estimate of their covariance matrix. However, the use of the two-step estimator in small samples, as in our study, has problem derived from proliferation of instruments. Furthermore, the estimated standard errors of the two-step GMM estimator tend to be small. Consequently, this paper employs the one-step GMM estimator.

In the specification, year dummies are used as instrument variable because other regressors are not strictly exogenous. The maximum lags length of independent variable which used as instrument is 2 to select the optimal lag, the AR(1) and AR(2) statistics are employed. There is convincing evidence that too many moment conditions introduce bias while increasing efficiency. It is, therefore, suggested that a subset of these moment conditions can be used to take advantage of the trade-off between the reduction in bias and the loss in efficiency. We restrict the moment conditions to a maximum of two lags on the dependent variable.

### Data and Empirical Strategy

We estimated Eq. (1) using the GMM estimator based on a panel of 33 OIC countries. Table S1 in File S1 lists the countries and their income groups in the sample. The choice of countries selected for this study is primarily dictated by availability of reliable data over the sample period among all OIC countries. The panel covers the period 1980–2008 and is unbalanced. Following [40], we use annual data in order to maximize sample size and to identify the parameters of interest more precisely. In fact, averaging out data removes useful variation from the data, which could help to identify the parameters of interest with more precision.

The dependent variable in our sample is logged per capita real GDP, using the purchasing power parity (PPP) exchange rates and is obtained from the Penn World Table (PWT 7.0). The economic dimension of KOF index is derived from Dreher et al. [41]. We use some other variables, along with economic globalization to control other factors influenced economic growth. Table S2 in File S2 shows the variables, their proxies and source that they obtain.

We relied on the three main approaches to capture the effects of economic globalization on economic growth in OIC countries. The first one is the baseline specification (Eq. (1)) which estimates the effect of economic globalization on economic growth.

The second approach is to examine whether the effect of globalization on growth depends on the complementary policies in the form of level of human capital and financial development. To test, the interactions of economic globalization and financial development (KOF*FD) and economic globalization and human capital (KOF*HCS) are included as additional explanatory variables, apart from the standard variables used in the growth equation. The KOF, HCS and FD are included in the model individually as well for two reasons. First, the significance of the interaction term may be the result of the omission of these variables by themselves. Thus, in that way, it can be tested jointly whether these variables affect growth by themselves or through the interaction term. Second, to ensure that the interaction term did not proxy for KOF, HCS or FD, these variables were included in the regression independently.

In the third approach, in order to study the role of income level of countries on the growth effect of globalization, the countries are split based on income level. Accordingly, countries were classified into three groups: high-income countries (3), middle-income (21) and low-income (9) countries. Next, dummy variables were created for high-income (Dum 3), middle-income (Dum 2) and low-income (Dum 1) groups. Then interaction terms were created for dummy variables and KOF. These interactions will be added to the baseline specification.

## Findings and Discussion

This section presents the empirical results of three approaches, based on the GMM -dynamic panel data; in Tables 1–3.Table 1 presents a preliminary analysis on the effects of economic globalization on growth. Table 2 displays coefficient estimates obtained from the baseline specification, which used added two interaction terms of economic globalization and financial development and economic globalization and human capital. Table 3 reports the coefficients estimate from a specification that uses dummies to capture the impact of income level of OIC countries on the growth effect of globalization.

**Table 1:** Baseline results.

Variables	Coefficient	t-statistics	*p*-value
**L1.GDP (Real GDP per capita, lag)**	0.13	1.93	0.054
**KOF (Economic globalization)**	0.67	3.85	0.000
**Government consumption (Government consumption %GDP)**	−0.0003	−0.09	0.92
**Inflation (Consumer prices index)**	−0.003	−0.89	0.37
**Human capital (School enrollment, secondary)**	0.0031	1.67	0.09
**Domestic investment (Gross capital formation % GDP)**	0.03	3.13	0.002
**Financial development (Liquid liabilities)**	0.055	3.16	0.002
**Institutional quality (ICRG Political risk index)**	−0.032	−0.34	0.73
**Countries**	33		
**Sargan Test**	0.45		
**First order serial correlation (** ***p*** **-value)**	0.000		
**Second order serial correlation (** ***p*** **-value)**	0.601		

Notes: Dependent variable: real GDP per capita in logarithm.

Cross-country panel data consisting of annual spanning 1980–2008.

Estimation method: Dynamic GMM estimator Arellano and Bond (1991).

**Table 2:** Economic globalization and Growth: Interaction terms.

Variables	Human capital	Financial development
**KOF**	0. 41(2.88)***	0.15 (0.86)
KOF *FD	-	0.001 (6.32)***
KOF *HC	0.002 (8.40)***	-
**Countries**	33	33
**Sargan Test (** ***p*** **-value)**	0.373	0.93
**First order serial correlation (** ***p*** **-value)**	0.000	0.000
**Second order serial correlation (** ***p*** **-value)**	0.115	0.387

Notes: Dependent variable: real GDP per capita in logarithm.

Cross-country panel data consisting of annual spanning 1980–2008.

Estimation method: Dynamic GMM estimator Arellano and Bond (1991).

A full set of year dummies is included to control for common time effects. The full regressions includes lagged of GDP per capita, government consumption, consumer price index, secondary school enrolment, gross capital formation, Liquid liability and ICRG.

Numbers below coefficients are the t-statistic.

***, **, *denotes statistical significance at the 1%, 5% and 10% levels, respectively.

**Table 3:** Growth effect of globalization at different income levels of countries.

Variables	Coefficient	t-statistics	*p*-value
**KOF**	0.88	4.35	0.000
KOF *dum1	−0.009	−3.08	0.002
KOF *dum2	0.003	2.02	0.043
KOF *dum3	0.045	2.55	0.011
**countries**	33		
**Sargan Test**	0.35		
**First order serial correlation (** ***p*** **-value)**	0.000		
**Second order serial correlation (** ***p*** **-value)**	0.152		

Notes: Dependent variable: real GDP per capita in logarithm. Cross-country panel data consisting of annual spanning 1980–2008.

Estimation method: Dynamic GMM estimator Arellano and Bond (1991).

A full set of year dummies is included to control for common time effects. The full regressions includes lagged of GDP per capita, government consumption, consumer price index, secondary school enrolment, gross capital formation, Liquid liability and ICRG.

The results in Table 1 indicate that economic globalization has positive impact on growth and the coefficient is significant at 1 percent level. The positive effect is consistent with the bulk of the existing empirical literature that support beneficial effect of globalization on economic growth [9], [11], [13], [19], [42], [43].

According to the theoretical literature, globalization enhances economic growth by allocating resources more efficiently as OIC countries that can be specialized in activities with comparative advantages. By increasing the size of markets through globalization, these countries can be benefited from economic of scale, lower cost of research and knowledge spillovers. It also augments capital in OICs as they provide a higher return to capital. It has raised productivity and innovation, supported the spread of knowledge and new technologies as the important factors in the process of development. The results also indicate that growth is enhanced by lower level of government expenditure, lower level of inflation, higher level of human capital, deeper financial development, more domestic investment and better institutions.

Table 2 represents that the coefficients on the interaction between the KOF, HCS and FD are statistically significant at 1% level and with the positive sign. The findings indicate that economic globalization not only directly promotes growth but also indirectly does via complementary reforms. On the other hand, the positive effect of economic globalization can be significantly enhanced if some complementary reforms in terms of human capital and financial development are undertaken.

In fact, the implementation of new technologies transferred from advanced economies requires skilled workers. The results of this study confirm the importance of increasing educated workers as a complementary policy in progressing globalization. However, countries with higher level of human capital can be better and faster to imitate and implement the transferred technologies. Besides, the financial openness brings along the knowledge and managerial for implementing the new technology. It can be helpful in improving the level of human capital in host countries. Moreover, the strong and well-functioned financial systems can lead the flow of foreign capital to the productive and compatible sectors in developing countries. Overall, with higher level of human capital and stronger financial systems, the globalized countries benefit from the growth effect of globalization. The obtained results supported by previous studies in relative to financial and trade globalization such as [5], [27], [44], [45].

Table (3) shows that the estimated coefficients on KOF*dum3 and KOF*dum2 are statistically significant at the 5% level with positive sign. The KOF*dum1 is statistically significant with negative sign. It means that increase in economic globalization in high and middle-income countries boost economic growth but this effect is diverse for low-income countries. The reason might be related to economic structure of these countries that are not received to the initial condition necessary to be benefited from globalization. In fact, countries should be received to the appropriate income level to be benefited by globalization.

The diagnostic tests in tables 1–3 show that the estimated equation is free from simultaneity bias and second-order correlation. The results of Sargan test accept the null hypothesis that supports the validity of the instrument use in dynamic GMM.

## Conclusions and Implications

Numerous researchers have investigated the impact of economic globalization on economic growth. Unfortunately, theoretical and the empirical literature have produced conflicting conclusions that need more investigation. The current study shed light on the growth effect of globalization by using a comprehensive index for globalization and applying a robust econometrics technique. Specifically, this paper assesses whether the growth effects of globalization depend on the complementary polices as well as income level of OIC countries.

Using a panel data of OIC countries over the 1980–2008 period, we draw three important conclusions from the empirical analysis. First, the coefficient measuring the effect of the economic globalization on growth was positive and significant, indicating that economic globalization affects economic growth of OIC countries in a positive way. Second, the positive effect of globalization on growth is increased in countries with higher level of human capital and deeper financial development. Finally, economic globalization does affect growth, whether the effect is beneficial depends on the level of income of each group. It means that economies should have some initial condition to be benefited from the positive effects of globalization. The results explain why some countries have been successful in globalizing world and others not.

The findings of our study suggest that public policies designed to integrate to the world might are not optimal for economic growth by itself. Economic globalization not only directly promotes growth but also indirectly does so via complementary reforms.

The policy implications of this study are relatively straightforward. Integrating to the global economy is only one part of the story. The other is how to benefits more from globalization. In this respect, the responsibility of policymakers is to improve the level of educated workers and strength of financial systems to get more opportunities from globalization. These economic policies are important not only in their own right, but also in helping developing countries to derive the benefits of globalization.

However, implementation of new technologies transferred from advanced economies requires skilled workers. The results of this study confirm the importance of increasing educated workers as a complementary policy in progressing globalization. In fact, countries with higher level of human capital can better and faster imitate and implement the transferred technologies. The higher level of human capital and certain skill of human capital determine whether technology is successfully absorbed across countries. This shows the importance of human capital in the success of countries in the globalizing world.

Financial openness in the form of FDI brings along the knowledge and managerial for implementing the new technology. It can be helpful in upgrading the level of human capital in host countries. Moreover, strong and well-functioned financial systems can lead the flow of foreign capital to the productive and compatible sectors in OICs.

In addition, the results show that economic globalization does affect growth, whether the effect is beneficial depends on the level of income of countries. High and middle income countries benefit from globalization whereas low-income countries do not gain from it. As Birdsall [46] mentioned globalization is fundamentally asymmetric for poor countries, because their economic structure and markets are asymmetric. So, the risks of globalization hurt the poor more. The structure of the export of low-income countries heavily depends on primary commodity and natural resource which make them vulnerable to the global shocks.

The major research limitation of this study was the failure to collect data for all OIC countries. Therefore future research for all OIC countries would shed light on the relationship between economic globalization and economic growth.

## Supporting Information

File S1 **Sample of Countries.**
(DOCX)

File S2 **The Name and Definition of Indicators.**
(DOCX)
