# Supplementary material for: Globalization and Economic Growth: Empirical Evidence on the Role of Complementarities
Source: PLoS One. 2014 Apr 10;9(4):e87824. doi: 10.1371/journal.pone.0087824 (PMC3982958; doi:10.1371/journal.pone.0087824)
Supplement: File S1 — Sample of Countries. (DOCX) [file pone.0087824.s001.docx]

**Table S1. Sample of Countries.**

| Low income | | |
| --- | --- | --- |
| Bangladesh | Guinea-Bissau | Sierra Leone |
| Burkina Faso | Mozambique | Togo |
| Guinea | Niger | Uganda |
| Middle income | | |
| Albania | Guyana | Nigeria |
| Algeria | Indonesia | Pakistan |
| Azerbaijan | Iran | Senegal |
| Cameroon | Jordan | Syrian Arab Republic |
| Côte d'Ivoire | Kazakhstan | Tunisia |
| Egypt | Malaysia | Turkey |
| Gabon | Morocco | Yemen |
| High income | | |
| Bahrain | Kuwait | Oman |
