# Supplementary material for: Globalization and Economic Growth: Empirical Evidence on the Role of Complementarities
Source: PLoS One. 2014 Apr 10;9(4):e87824. doi: 10.1371/journal.pone.0087824 (PMC3982958; doi:10.1371/journal.pone.0087824)
Supplement: File S2 — The Name and Definition of Indicators. (DOCX) [file pone.0087824.s002.docx]

**Table S2. The Name and Definition of Indicators.**

|  | Indicator | Source of data |
| --- | --- | --- |
| GDP | PPP Converted GDP Per Capita | The Penn World Tables (PWT 7.0) |
| GINI | The Standardized World Income Inequality Database (SWIID) | [[47](#_ENREF_47)] |
| KOF | Economic dimension of KOF globalization index | KOF website  [[41](#_ENREF_41)] |
| CG | Government consumption | The penn world tables (pwt 7.0) |
| INVG | Gross capital formation (% of GDP) | World Development Indicators 2009 |
| CPI | Inflation, consumer prices (annual %) | World Development Indicators 2009 |
| HCS | School enrollment, secondary (% gross) | World Development Indicators 2009 |
| FD | Financial development ( liquid liabilities / GDP) | [[48](#_ENREF_48)] |
| ICRG | Institutional quality | International Country Risk Guide |
